# Energetic Stability and Band-Edge Orbitals of Layered Inorganic Perovskite Compounds for Solar Energy Applications

**DOI:** 10.1021/acs.jpcc.3c04528

**Published:** 2023-10-06

**Authors:** M. Oluchi Anunobi, Robert F. Berger

**Affiliations:** Department of Chemistry, Western Washington University, Bellingham, Washington 98225-9150, United States

## Abstract

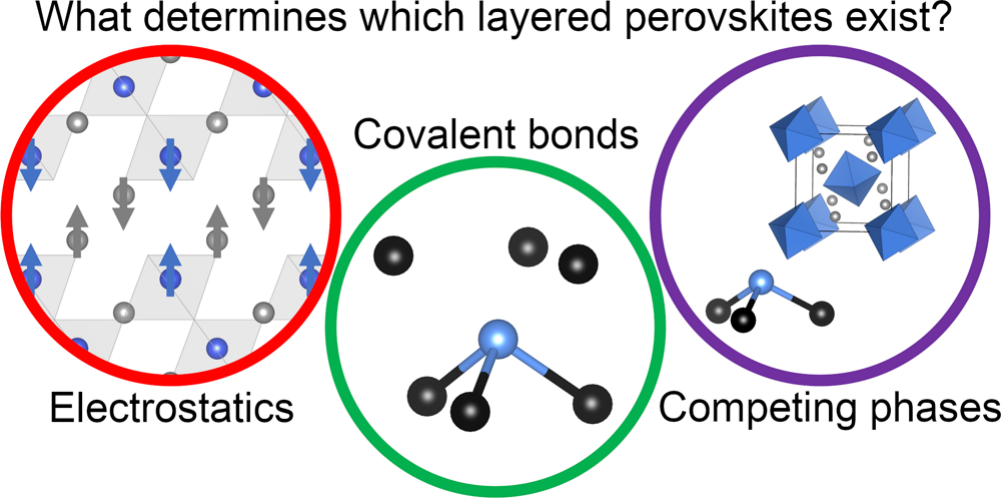

Halide and oxide perovskite semiconductors (e.g., CsPbI_3_ and SrTiO_3_) have been widely studied for solar energy conversion applications. The optoelectronic properties and performance of these compounds can be tuned through the growth of layered perovskite superstructures. While oxides are quite varied in the compositions and geometries taken up by layered perovskites, halides have proven much more limited. In this paper, we use density functional theory calculations and chemical intuition to explore why this is the case. We show that, in general, the thermodynamic stability or instability of layered perovskite superstructures depends on the interplay of their ionic and covalent character and, just as importantly, on the features of other competing phases.

## Introduction

Since the 2009 work of Miyasaka and co-workers on CH_3_NH_3_PbI_3_ and CH_3_NH_3_PbBr_3_,^1^ lead halide perovskite compounds have generated great interest as light absorbers for photovoltaic applications.^2−6^ More broadly, the class of perovskite semiconductors (general formula ABX_3_) used in solar energy conversion also includes oxide photocatalysts such as SrTiO_3_.^7−11^ The rise of perovskites in recent years is due in part to their remarkable tunability in composition and structure. Halide perovskites can be doped and substituted at all crystallographic sites. A-site cations may be either organic (e.g., CH_3_NH_3_^+^) or inorganic (e.g., Cs^+^), B-site cations are typically Pb^2+^ but may be substituted with other elements or combinations thereof (e.g., Sn^2+^ or Ag^+^/Bi^3+^), and X-site anions may be any halides or mixtures thereof. Oxide perovskite photocatalysts are also composed of a variety of A- and B-site cations, typically with d^0^ B-site cations such as Ti^4+^ or Nb^5+^.

**Figure 1:**
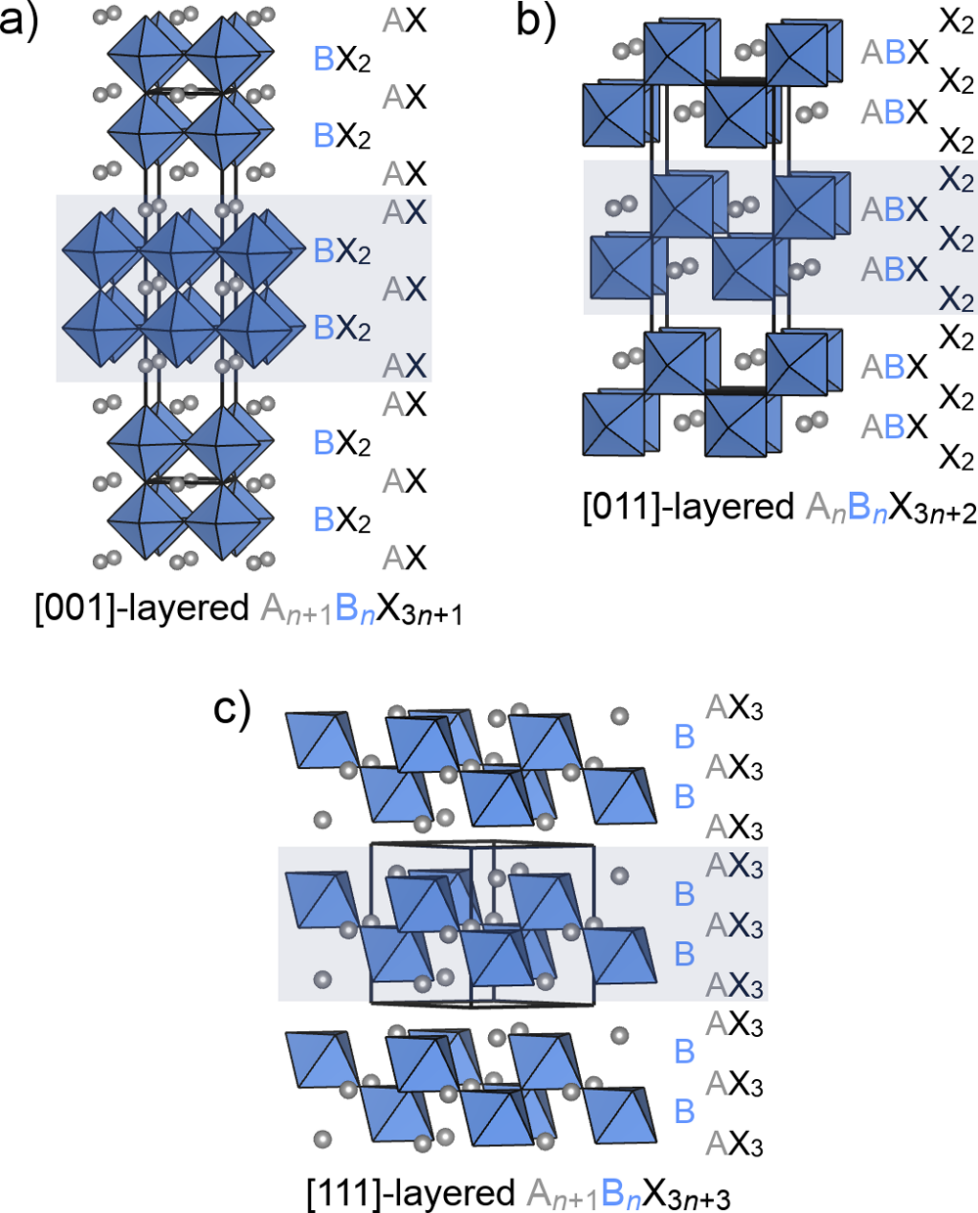
Examples of layered superstructures in which perovskite regions are aligned in various directions. (a) Ruddlesden–Popper phases (A_*n*+1_B_*n*_X_3*n*+1_) are layered in the [001] direction. (b) Compounds layered in the [011] direction have the general formula A_*n*_B_*n*_X_3*n*+2_. (c) Compounds layered in the [111] direction have the general formula A_*n*+1_B_*n*_X_3*n*+3_. All images in this figure show *n* = 2 phases, with a two-layer perovskite region highlighted in gray and the composition of each atomic layer labeled.

Beyond elemental substitution of ABX_3_ compounds, perovskite materials and their properties can be further tuned through the growth of layered phases in which blocks of the ABX_3_ structure are separated by additional spacer layers.^12−15^ Well-known examples of this type of layering are the Ruddlesden–Popper (RP) phases,^16,17^ which exist experimentally for combinations of elements such as Sr–Ti–O. RP phases (Figure 1a) have the general formula A_*n*+1_B_*n*_X_3*n*+1_ (*n* = 1, 2, 3, ...) and are built from *n*-layer perovskite blocks spaced by an additional AX layer perpendicular to the cubic perovskite unit cell axis (the [001] direction). Analogous series exist in which perovskite blocks are layered along other high-symmetry axes ([011] and [111], Figure 1b,c), as will be described later in this paper.

While these series of layered perovskites have clear patterns in their structure, the question of which of these phases are thermodynamically stable is more difficult to predict, especially in inorganic halide perovskites. For example, while many oxide RP phases have been synthesized experimentally, inorganic halide RP phases analogous to perovskite photovoltaics are rare. (The halide RP phases that have been synthesized have mixed occupancy at the X-sites^18,19^ and/or perovskite blocks separated by organic linkers rather than inorganic cations.^12−14,20−24^) Instead, experimental examples of layering in inorganic halide perovskites are observed almost exclusively in the [111] direction.^25−28^ This is in sharp contrast to layered d^0^ oxide perovskites, for which there are numerous examples of compounds layered in the [001], [011], and [111] directions.^29−31^

The stability or instability of a layered perovskite compound depends on a delicate interplay of requirements for charge balance, compatibility of ionic sizes, and questions about the energetic favorability of competing phases. This challenge of predicting the stability of a series of layered perovskites makes it more difficult for synthetic chemists and materials scientists to discover, design, and tune such materials. In this paper, we use density functional theory (DFT) calculations and chemical intuition to explore the factors governing the thermodynamic stability of inorganic layered perovskites as well as features of their band-edge orbitals. In doing so, we find that both the ionic and covalent characteristics of these compounds are key in determining which phases are stable.

## Computational Methods

**Figure 2:**
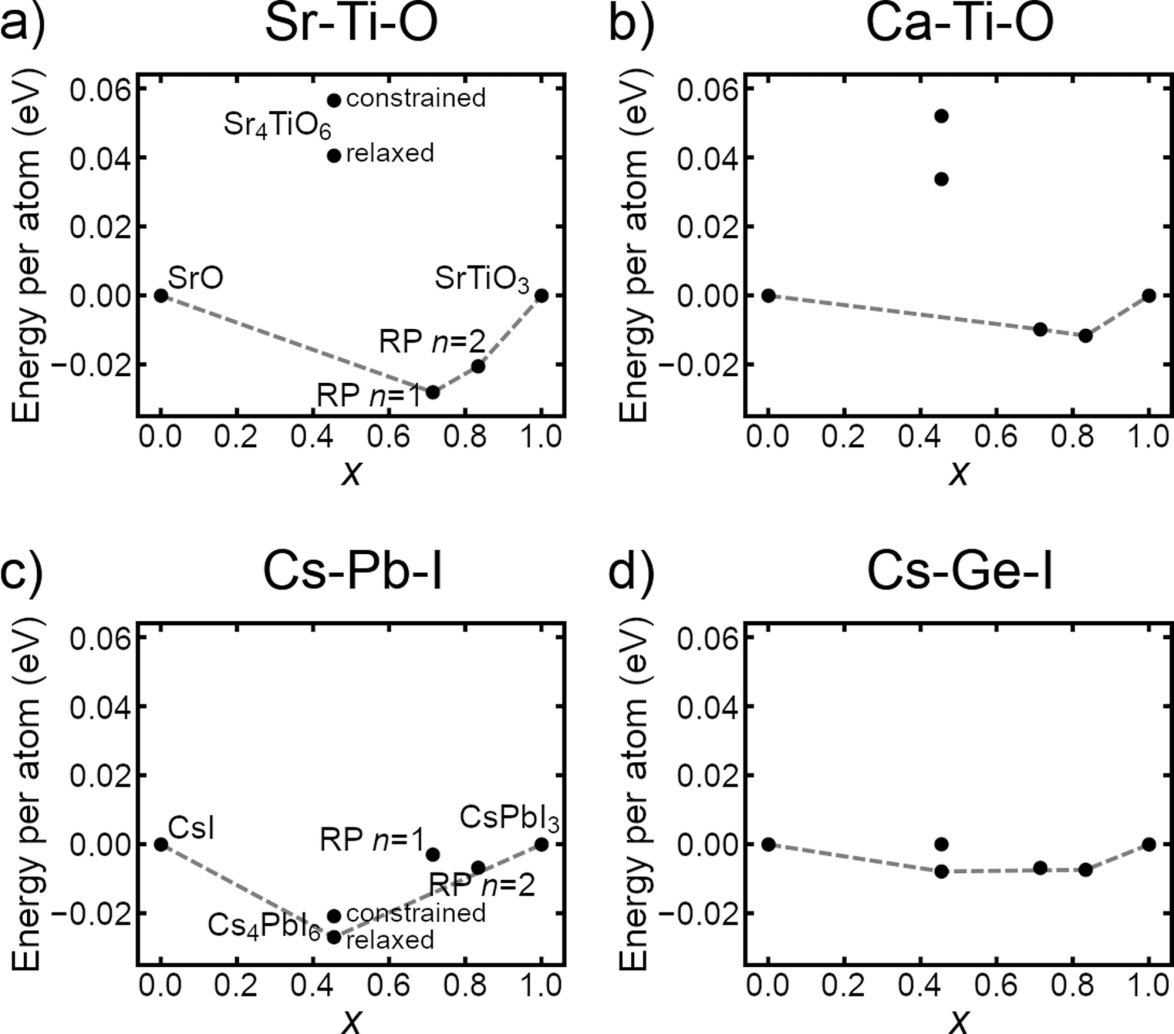
Comparisons of the structural energy per atom of phases within the (a) Sr–Ti–O, (b) Ca–Ti–O, (c) Cs–Pb–I, and (d) Cs–Ge–I systems. Phases compared are NaCl-type AX, A_4_BX_6_ (fully relaxed and with B–X bonds constrained to the corresponding cubic perovskite bond lengths), RP with *n* = 1 and *n* = 2, and cubic perovskite ABX_3_. Zero energy is defined as the energy of a combination of AX and ABX_3_. Dashed gray lines show the convex hull formed by the lowest-energy phases. The stoichiometric parameter *x* is defined as the fraction of ABX_3_ atoms when a compound is expressed as a sum of AX and ABX_3_.

Structural geometries and energies in this paper are computed within density functional theory (DFT) using the VASP package^32−35^ and PAW potentials^36^ (details in the Supporting Information). The PBE functional, a generalized gradient approximation, is employed for its proven ability to capture the relative energies of competing phases.^37^ To consider the possibility that van der Waals forces affect the reported trends, we provide in the Supporting Information results analogous to Figure 2a–d in which van der Waals corrections are included using the D3 method of Grimme.^38,39^ We find that relative structural energies with and without van der Waals corrections are always within 0.006 eV/atom of each other and that the overall trends remain unchanged. We therefore report results without van der Waals corrections throughout the text of the paper.

Each crystal structure is optimized with a fixed space group. Though perovskite-derived compounds often undergo symmetry-lowering distortions (e.g., octahedral rotations in halide perovskites) that would lower their DFT-computed energies slightly, we focus on high-symmetry phases in order to remain consistent across compounds and to control for complicating variables. Structures are therefore optimized with the following space groups: NaCl-type AX , cubic perovskite ABX_3_, [001]-layered A_2_BX_4_ and A_3_B_2_X_7_ (*I*4/*mmm*), [011]-layered ABX_4_ (*Cmcm*), [111]-layered A_3_B_2_X_9_ (*P*3̅*m*1), and A_4_BX_6_ (*R*3̅*c*).

**Figure 3:**
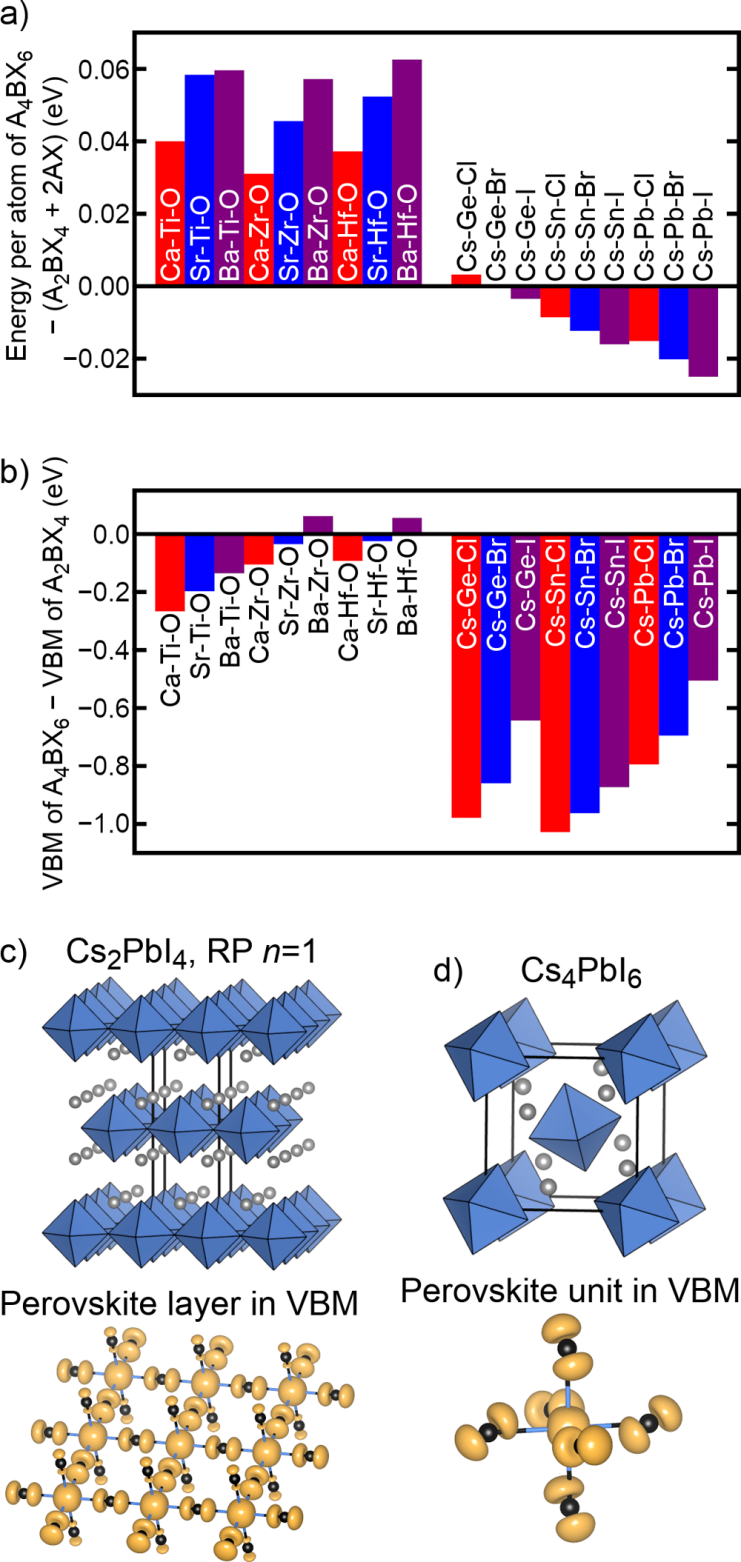
(a) Differences in the structural energy per atom of A_4_BX_6_ phases and combinations of the respective *n* = 1 Ruddlesden–Popper (RP) phases and NaCl-type AX phases. (b) Differences between the absolute energies of the valence band maxima (VBM) of A_4_BX_6_ phases and the respective *n* = 1 RP phases. The differing dimensionality of perovskite regions (top) and VBM electron densities (bottom) help to explain why (c) halide RP phases have significantly higher-energy VBM than (d) halide A_4_BX_6_ phases.

Calculations of 5-atom cubic perovskite unit cells use a Γ-centered 6 × 6 × 6 *k*-point mesh, while calculations of perovskite superstructures use proportionally fewer *k*-points. A plane-wave basis set cutoff of 450 eV is used throughout. In cases where absolute band-edge energies are discussed, we leverage the fact that the lowest-energy band in a calculation is quite flat and is effectively an atomic orbital whose energy can serve as a reference across related compounds. Therefore, the absolute energy of a band edge is taken to be its energy *above the lowest-energy band* at *k*-point Γ = (0, 0, 0). This enables fair comparisons between the band-edge energies of related compounds (e.g., A_4_BX_6_ and A_2_BX_4_ for the same elements). To consider the possibility that spin–orbit coupling affects the reported trends, we provide in the Supporting Information a subset of results analogous to Figure 3b in which spin–orbit coupling is included. We find that band-edge energy differences between related compounds with and without spin–orbit coupling are always within 0.1 eV of each other and that the overall trends remain unchanged. We therefore report results without spin–orbit coupling throughout the body of the paper.

Bader charges on the atoms are computed in VASP as implemented by Henkelman and co-workers.^40^ The structures of existing compounds are identified with the aid of the Materials Project database.^41^ Images of crystal structures in this work are produced using the software VESTA.^42^

## Series of Inorganic Layered Perovskites

### Layering in Various Crystallographic Directions

The series of layered perovskite phases we consider in this article (Figure 1a–c) all consist of blocks of the ABX_3_ perovskite structure spaced by additional B-deficient layers. The exact geometries and stoichiometries of these phases depend on the directions in which they are layered. When the perovskite structure is viewed along [001], it consists of alternating AX and BX_2_ layers. When *n* perovskite formula units are spaced by an additional AX layer, the resulting stoichiometry is (ABX_3_)_*n*_(AX) = A_*n*+1_B_*n*_X_3*n*+1_—that is, the RP phases (Figure 1a). When the perovskite structure is viewed along [011], it consists of alternating X_2_ and ABX layers. When *n* perovskite formula units are spaced by an additional X_2_ layer, the resulting stoichiometry is (ABX_3_)_*n*_(X_2_) = A_*n*_B_*n*_X_3*n*+2_ (Figure 1b). Finally, when the perovskite structure is viewed along [111], it consists of alternating AX_3_ and B layers. When *n* perovskite formula units are spaced by an additional AX_3_ layer, the resulting stoichiometry is (ABX_3_)_*n*_(AX_3_) = A_*n*+1_B_*n*_X_3*n*+3_ (Figure 1c). To varying degrees, members of all of these series have been synthesized and discussed in the literature.

To make the essentially infinite compositional parameter space of layered perovskites more manageable, we focus on ternary, fully inorganic compounds in these three series. In other words, we will not focus on compounds with mixed occupancy or polyatomic organic cations, which further contribute to the compositional richness of this class of materials. In one sense, these requirements that compounds be inorganic and ternary may be seen as limiting the scope of this work as they rule out many technologically relevant compounds. However, this narrowed scope may also be viewed as an opportunity. By focusing on compounds that are the structurally simplest representatives of a broader class, we may elucidate energetic driving forces that govern *all* layered perovskites, including those with more complex composition and symmetry. The features we highlight are based on linear combinations of atomic orbitals and are therefore relevant even as synthetic scientists mix and replace ions in ingenious ways.

### Requirements for Charge Balance and Closed-Subshell Ions

Inorganic perovskite compounds used in solar energy conversion (e.g., SrTiO_3_ and CsPbI_3_) are generally viewed as ionic. Their ability to absorb and convert sunlight stems from the fact that they are semiconductors, consisting of closed-shell or closed-subshell ions. In Sr^2+^Ti^4+^O_3_^2–^, all three types of ions have noble gas electron configurations, resulting in a band gap between the filled oxide 2p states and the unfilled titanium 3d states. In Cs^+^Pb^2+^I_3_^–^, the A- and X-sites have noble gas electron configurations while the B-site has a filled s^2^ subshell, resulting in a band gap between filled iodide 5p/lead 6s states and unfilled lead 6p states.

We are interested in exploring layered perovskites with the potential to absorb and convert sunlight in an analogous manner. We therefore focus on layered compounds with the same charge balance of ions with closed subshells. In some cases, a charge balance of ions with closed subshells is possible with the same combinations of elements that form perovskites. For example, if the A- and X-site charges are equal and opposite, then [001]-layered RP phases may exist for the same elements and ionic charges as perovskites. Therefore, perovskites of the form A^2+^B^4+^X_3_^2–^ (e.g., SrTiO_3_) and A^+^B^2+^X_3_^–^ (e.g., CsPbI_3_) have the appropriate charges to form RP phases A_*n*+1_B_*n*_X_3*n*+1_. However, other stable perovskites such as K^+^Ta^5+^O_3_^2–^ and La^3+^Al^3+^O_3_^2–^ do not have the appropriate charge balance to form RP phases with the same ions.

In contrast, layering in the [011] and [111] directions never allows charge-balanced compounds to form from the same ions as charge-balanced ABX_3_ perovskites. Instead, charge balance can be achieved for different combinations of elements layered in the [011] and [111] directions. For example, La_2_^3+^Ti_2_^4+^O_7_^2–^, Sr_2_^2+^Nb_2_^5+^O_7_^2–^, La_4_^3+^Ti_3_^4+^O_12_^2–^, Sr_5_^2+^Nb_4_^5+^O_15_^2–^, and Cs_3_^+^Bi_2_^3+^Br_9_^–^ are all existing, charge-balanced, layered perovskite superstructures whose ionic charges are not suitable for ABX_3_ perovskites.

Tables 1 and 2 enumerate the many reasonable combinations of A- and B-site ionic charges that lead to charge balance in perovskites layered in the [001], [011], and [111] directions. To keep the compositional parameter space manageable, we apply several constraints: (1) The compounds are ternary (with no mixed occupancy at any site) and inorganic. (2) The X-site is occupied by oxide, sulfide, or halide anions. (3) The A- and B-sites are occupied by cations of integer charge. (4) The charge on A is less than or equal to the charge on B, which is less than or equal to 6+. (5) The perovskite blocks are wide enough to form connected networks (which rules out *n* = 1 for compounds layered along [011] and [111]).

**Table 1:** Combinations of A- and B-Site Ionic Charges That Result in Layered, Charge-Balanced Oxide or Sulfide Perovskites (X^2–^)^a^

[001] layering (A_*n*+1_B_*n*_X_3*n*+1_)		[011] layering (A_*n*_B_*n*_X_3*n*+2_)		[111] layering (A_*n*+1_B_*n*_X_3*n*+3_)	
A_2_BX_4_ (*n* = 1)	+/6+				
	**2**+/**4**+				
A_3_B_2_X_7_ (*n* = 2)	**2**+/**4**+	ABX_4_ (*n* = 2)	2+/6+	A_3_B_2_X_9_ (*n* = 2)	2+/6+
			**3**+/**5**+		
			4+/4+		
A_4_B_3_X_10_ (*n* = 3)	**2**+/**4**+	A_3_B_3_X_11_ (*n* = 3)	none	A_4_B_3_X_12_ (*n* = 3)	**3**+/**4**+
A_5_B_4_X_13_ (*n* = 4)	**2**+/**4**+	A_2_B_2_X_7_ (*n* = 4)	+/**6**+	A_5_B_4_X_15_ (*n* = 4)	**2**+/**5**+
			**2**+/**5**+		
			**3**+/**4**+		
A_6_B_5_X_16_ (*n* = 5)	**2**+/**4**+	A_5_B_5_X_17_ (*n* = 5)	none	A_6_B_5_X_18_ (*n* = 5)	+/6+

a Possibilities are limited by constraints described in the text, pairs of charges are listed with A-site followed by B-site charge, and bold text indicates that at least one such compound has been observed experimentally according to the Materials Project Database.^41^

**Table 2:** Combinations of A- and B-Site Ionic Charges That Result in Layered, Charge-Balanced Halide Perovskites (X^–^)^a^

[001] layering (A_*n*+1_B_*n*_X_3*n*+1_)		[011] layering (A_*n*_B_*n*_X_3*n*+2_)		[111] layering (A_*n*+1_B_*n*_X_3*n*+3_)	
A_2_BX_4_ (*n* = 1)	+/2+				
A_3_B_2_X_7_ (*n* = 2)	+/2+	ABX_4_ (*n* = 2)	+/3+	A_3_B_2_X_9_ (*n* = 2)	+/**3**+
			2+/2+		
A_4_B_3_X_10_ (*n* = 3)	+/2+	A_3_B_3_X_11_ (*n* = 3)	none	A_4_B_3_X_12_ (*n* = 3)	none
A_5_B_4_X_13_ (*n* = 4)	+/2+	A_2_B_2_X_7_ (*n* = 4)	none	A_5_B_4_X_15_ (*n* = 4)	none
A_6_B_5_X_16_ (*n* = 5)	+/2+	A_5_B_5_X_17_ (*n* = 5)	none	A_6_B_5_X_18_ (*n* = 5)	none

a Possibilities are limited by constraints described in the text, pairs of charges are listed with A-site followed by B-site charge, and bold text indicates that at least one such compound has been observed experimentally according to the Materials Project Database.^41^

It is clear from Tables 1 and 2 that both oxides and halides could, based on charge balance alone, plausibly exist as perovskite-derived compounds layered in the [001], [011], and [111] directions. However, only some of these combinations of charges (those shown in bold in Tables 1 and 2) actually form experimentally.

### Layered Perovskites Known to Exist Experimentally

Based on phases listed as “experimentally observed” in the Materials Project Database,^41^Tables 3 and 4 show oxide and halide compounds known to crystallize as layered ternary perovskite superstructures. In some cases, these compounds undergo the same types of B–X octahedral rotations as ABX_3_ perovskites themselves. Given our focus on solar energy conversion applications, these tables are not a complete accounting of layered perovskites. Instead, Tables 3 and 4 show only compounds that are isoelectronic with d^0^ oxide photocatalysts (e.g., SrTiO_3_) and s^2^ halide photovoltaics (e.g., CsPbI_3_)—that is, the compounds with band gaps and band-edge orbitals most likely to be amenable to solar energy conversion. Other layered perovskites (e.g., hydrides, compounds whose cations have partially filled d orbitals, and fluorides with main-group B-site cations) are not shown.

**Table 3:** Layered Oxide and Sulfide Perovskites with d^0^ B-Site Cations That Have Been Observed Experimentally According to the Materials Project Database^41^ a

[001] layering (A_*n*+1_B_*n*_X_3*n*+1_)		[011] layering (A_*n*_B_*n*_X_3*n*+2_)	[111] layering (A_*n*+1_B_*n*_X_3*n*+3_)
*n* = 1	Sr_2_TiO_4_, Ba_2_ZrO_4_,		
	Ba_2_ZrS_4_, Ba_2_HfS_4_		
*n* = 2	Ca_3_Ti_2_O_7_, Sr_3_Ti_2_O_7_,	LnTaO_4_	none
	Ba_3_Zr_2_S_7_		
*n* = 3	Ca_4_Ti_3_O_10_, Sr_4_Ti_3_O_10_,	none	Ln_4_Ti_3_O_12_
	Ba_4_Zr_3_S_10_, Ba_4_Hf_3_S_10_		
*n* = 4	Ba_5_Hf_4_S_13_	Na_2_W_2_O_7_, Ca_2_Nb_2_O_7_,	Ba_5_Nb_4_O_15_, Ln_5_Nb_4_O_15_,
		Sr_2_Nb_2_O_7_, Sr_2_Ta_2_O_7_,	Ba_5_Ta_4_O_15_, Ln_5_Ta_4_O_15_
		Ln_2_Ti_2_O_7_	
*n* = 5	Ba_6_Hf_5_S_16_	none	none

a Lanthanide elements are grouped together as “Ln”.

**Table 4:** Layered Halide Perovskites with s^2^ B-Site Cations That Have Been Observed Experimentally According to the Materials Project Database^41^ a

[001] layering (A_*n*+1_B_*n*_X_3*n*+1_)		[011] layering (A_*n*_B_*n*_X_3*n*+2_)	[111] layering (A_*n*+1_B_*n*_X_3*n*+3_)
*n* = 1	none		
*n* = 2	none	none	Cs_3_As_2_Cl_9_, Rb_3_Sb_2_Br_9_,
			Cs_3_Sb_2_Cl_9_, Cs_3_Sb_2_Br_9_,
			Cs_3_Sb_2_I_9_, Cs_3_Bi_2_Cl_9_,
			Cs_3_Bi_2_Br_9_
*n* = 3	none	none	none
*n* = 4	none	none	none
*n* = 5	none	none	none

a Lanthanide elements are grouped together as “Ln”.

Several trends are clear in Tables 3 and 4. In oxides layered in all directions ([001], [011], and [111]), most combinations of charges that are balanced are actually observed. The few that are not observed are those with very large positive charges (4+ at the A-site or 6+ at the B-site), which unsurprisingly take on structures with lower coordination for their small cations. Layered oxide perovskites have such structural flexibility that phases layered in multiple directions may form from the same elements when charge balance allows (e.g., Ln_2_Ti_2_O_7_ and Ln_4_Ti_3_O_12_). In halides, there is a relative lack of variety in layered inorganic s^2^ halide perovskites. There are no ternary inorganic halide RP phases (though halide RP phases have been synthesized with mixed occupancy at the X-site^18,19^ and with organic linkers at the A-site^13,20−24^), even though such compounds would be charge-balanced. Furthermore, when layering in either the [011] or [111] direction is allowed by charge balance, halide compounds consistently prefer phases layered in the [111] direction with the stoichiometry A_3_B_2_X_9_.

For the remainder of this article, we aim to rationalize these trends in energetic stability. Considering clues in structural distortions, ionic charges, and the character of the highest-energy filled crystal orbitals, we find that the structural differences between layered oxide and halide perovskites stem not only from features of those compounds themselves but also of other phases with which the layered perovskites compete.

## Results and Discussion

### Lack of Inorganic Halide Ruddlesden–Popper (RP) Phases

As shown in Tables 3 and 4, there are many experimentally observed d^0^ oxide RP phases, but a notable lack of inorganic s^2^ halide RP phases. This is not due to any issues with charge balance, as the same combinations of elements that form s^2^ halide perovskites (e.g., Cs–Pb–I) could form charge-balanced RP phases. Why, then, do these halide RP phases tend not to form?

To begin to rationalize this observation, we must consider which other phases may be energetically competitive with the RP phases. In other words, whether a compound is “stable” or “unstable” depends not just on its own features but on the features of competing arrangements of the same elements. One natural possibility is that A_*n*+1_B_*n*_X_3*n*+1_ RP phases are in energetic competition with their structural building blocks, AX + *n*ABX_3_. Another important competing phase is the A_4_BX_6_ structure (Figure 3d), which can accommodate the same ionic charges as oxide and halide combinations of elements such as Sr–Ti–O and Cs–Pb–I, and is observed experimentally in halides including Cs_4_PbCl_6_ and Cs_4_PbBr_6_.^43,44^ The A_4_BX_6_ structure can be viewed as isolated perovskite units that each resemble a cubic perovskite unit cell but whose B–X octahedra are not connected in a corner-sharing network.

For both oxide and halide combinations of elements, Figure 2a–d compares the computed energies per atom of the *n* = 1 and *n* = 2 RP phases, NaCl-type AX phases, cubic perovskite phases, and A_4_BX_6_ phases. It would be helpful to first clarify what is meant by “energy per atom”. The chemical formula of each compound in this figure can be expressed as the sum of AX and ABX_3_. We define *x* as the fraction of atoms in a compound that are in the ABX_3_ portion (as opposed to the AX portion). For example, A_4_BX_6_ can be written as 3(AX) + ABX_3_, meaning  of its atoms are in the ABX_3_ portion. The “energy per atom” of a compound in Figure 2a–d is defined as *E*(compound) – [(1 – *x*)*E*(AX) + *xE*(ABX_3_)], where each *E* is a DFT-computed total energy divided by the number of atoms in the respective unit cell. Note that using these conventions, the energies of the end points AX and ABX_3_ are zero by definition, while a compound with negative energy is more stable than separate AX and ABX_3_ phases. One can infer that compounds on the straight lines connecting the lowest-energy phases (the convex hull) are expected to be thermodynamically stable at a low temperature. The figure shows that the RP phases for both oxides (Figure 2a,b) and halides (Figure 2c,d) are in close competition with their building blocks, AX + *n*ABX_3_. However, the notable difference is that A_4_BX_6_ generally outcompetes 3(AX) + ABX_3_ for halides but not for oxides.

Figure 3a further illustrates, for a wider range of elements, the preference of d^0^ oxides for RP phases and s^2^ halides for the A_4_BX_6_ phase. The positive energy differences for d^0^ oxides in Figure 3a indicate that A_4_BX_6_ oxides are significantly less stable than 2(AX) + A_2_BX_4_, while the negative energy differences for nearly all s^2^ halides indicate that A_4_BX_6_ halides are more stable than 2(AX) + A_2_BX_4_. Possible reasons for these trends can be classified as steric or electronic. The steric suitability of a combination of elements for perovskite formation is often discussed in terms of Goldschmidt tolerance factors.^45−48^ When the ionic radii yield a value of , they pack comfortably as hard spheres at mutually compatible bond distances. Because inorganic halide perovskites (even those with A = Cs^+^) tend to have tolerance factors well below 1, one might think the A_4_BX_6_ phase could be a favorable arrangement for halides in which A–X and B–X bond lengths are not constrained to the same corner-sharing octahedral network as perovskites and RP phases. While this explanation is intuitively plausible, it can be tested and largely dismissed by constraining the B–X bond lengths within the A_4_BX_6_ structure. Figure 2a–d shows that even when B–X bond distances are artificially shortened to match the lengths in the corresponding cubic perovskites, the A_4_BX_6_ phase continues to be much more energetically competitive with RP phases for halides than for oxides. Furthermore, this observation remains true even when comparing oxide Ca–Ti–O and halide Cs–Ge–I, which have similar tolerance factors (0.97 and 0.98, respectively, based on Shannon crystal radii^49^).

Having ruled out a tolerance-factor-based argument for the differing stability of oxide and halide RP phases, we now focus on the electronic differences between the two classes of compounds. Because the B-site cations in an s^2^ halide compound have a lone electron pair that those in a d^0^ oxide compound lack, halides and oxides differ significantly in their band-edge orbital character. We focus here on the energy and character of the valence band maxima (VBM) of various compounds, which tend to correlate strongly with structural stability. As shown in Figure 3b, the computed VBM of halide A_4_BX_6_ compounds are generally 0.5–1.0 eV lower in energy than their *n* = 1 RP counterparts, while the VBM of oxide A_4_BX_6_ compounds are generally within 0.3 eV of their *n* = 1 RP counterparts. These differences can be understood by viewing the character of these crystal orbitals. In halides, the highest-energy filled orbitals have antibonding interactions between nearest neighbors (B-site s and X-site p). This makes the energy of these orbitals higher for a connected corner-sharing network (as in a RP phase, Figure 3c) in which each X-site anion is bonded to two B-site cations than for an isolated octahedron (as in A_4_BX_6_, Figure 3d) in which each X-site anion is bonded to only one B-site cation. In oxides, however, the highest-energy filled orbitals consist of X-site p states that are not nearest neighbors, making their energies less sensitive to octahedral connectivity. These ideas help to explain why, although RP phases are in similarly close competition with their corresponding AX and perovskite ABX_3_ phases for both oxides and halides, the A_4_BX_6_ phase outcompetes RP phases only for halides.

### Prevalence of [111]-Layered Halide Perovskites

A second key observation from Tables 3 and 4 is that there are many perovskite-derived inorganic s^2^ halide compounds layered in the [111] direction, but none layered in the [011] direction. (As with the RP phases, there do exist [011]-layered s^2^ halide compounds with organic linkers at the A-site.^12,14,50,51^) This is in contrast to d^0^ oxides, for which both orientations are commonly observed. As explained earlier, this is not an issue of charge balance in halides, but of one orientation energetically outcompeting the other. For example, while the (Rb, Cs)–(As, Sb, Bi)–(Cl, Br, I) systems could potentially form [011]-layered ABX_4_ or [111]-layered A_3_B_2_X_9_ phases, they exclusively form A_3_B_2_X_9_ experimentally. As in the previous section, in order to understand why [011]-layered oxide perovskites exist, while [011]-layered halide perovskites do not, we must consider not only the compounds themselves but also the other phases they compete with.

**Figure 4:**
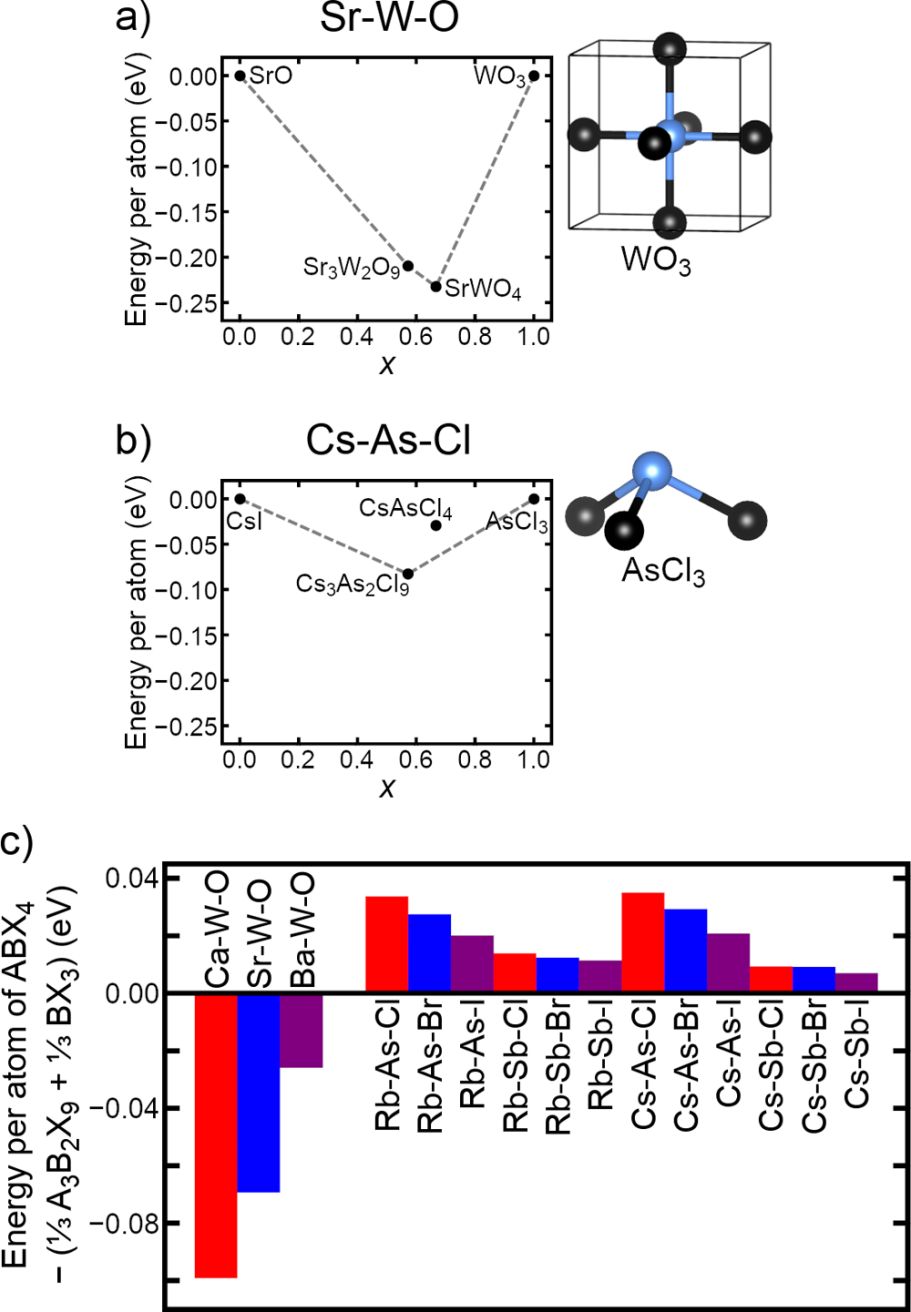
Comparisons of the structural energy per atom of phases within the (a) Sr–W–O and (b) Cs–As–Cl systems. Phases compared are NaCl-type AX, [111]-layered A_3_B_2_X_9_, [011]-layered ABX_4_, and BX_3_ (pictures of which are shown in the respective panels). Zero energy is defined as the energy of a combination of AX and BX_3_. Dashed gray lines show the convex hull formed by the lowest-energy phases. The stoichiometric parameter *x* is defined as the fraction of BX_3_ atoms when a compound as expressed as a sum of AX and BX_3_. (c) Differences in the structural energy per atom of ABX_4_ phases and combinations of the respective A_3_B_2_X_9_ and BX_3_ phases are shown for a variety of combinations of elements.

The DFT-PBE calculations shown in Figure 4a–c help us to see the complete picture. The figure focuses on two combinations of elements, Sr–W–O and Cs–As–Cl—an oxide and a halide that could form charge-balanced [011]-layered ABX_4_ and [111]-layered A_3_B_2_X_9_ phases. (While the Sr–W–O system experimentally prefers phases with tetrahedrally coordinated W^6+^ cations due to their small ionic radius, this proves a useful example to illustrate the contrast between oxides and halides.) Figure 4a shows that in the compositional space between binary phases SrO and WO_3_, both Sr_3_W_2_O_9_ and SrWO_4_ are on the convex hull, suggesting the possible existence of both phases (when competing phases with tetrahedral coordination are not considered). In contrast, Figure 4b shows that in the compositional space between binary phases CsCl and AsCl_3_, only Cs_3_As_2_Cl_9_ but not CsAsCl_4_ is on the convex hull, suggesting that only the [111]-layered perovskite is likely to exist.

The crucial difference between the oxide and halide systems in this case is not so much in the layered perovskites themselves but rather in the binary BX_3_ compounds with which those layered perovskites compete. In the case of the oxide, WO_3_ is essentially a perovskite with a vacant A-site (shown in Figure 4a), meaning that its octahedral coordination is similar to the layered perovskites. In contrast, AsCl_3_ has an entirely different structure, that of a covalently bonded trigonal pyramidal molecule (shown in Figure 4b). This radically changes the energetic competition within the Cs–As–Cl system, moving CsAsCl_4_ well above the convex hull due to the high stability of AsCl_3_. Figure 4c shows that this trend holds for a range of elements. For (Ca, Sr, Ba)–W–O oxides, the negative energies indicate that [011]-layered AWO_4_ is more stable than separate A_3_W_2_O_9_ and WO_3_ phases. For (Rb, Cs)–(As, Sb)–(Cl, Br, I) halides, the positive energies indicate that [011]-layered ABX_4_ is less stable than separate A_3_B_2_X_9_ and BX_3_ phases and is therefore unlikely to exist. Note that for simplicity AsX_3_ and SbX_3_ are calculated as gas-phase molecules, though they exist as molecular crystals below their melting point.

**Figure 5:**
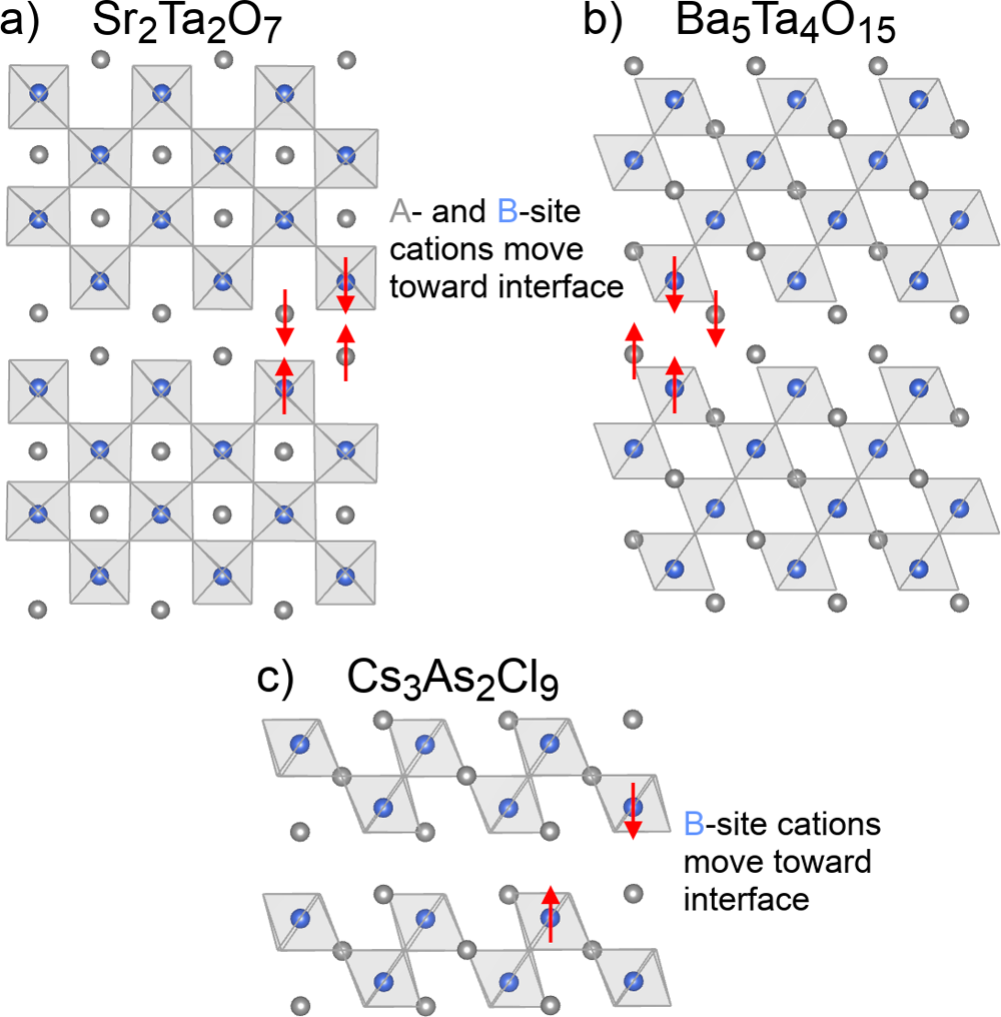
Illustrations of the movements of cations toward the interfaces between perovskite blocks in the layered perovskite phases (a) Sr_2_Ta_2_O_7_, (b) Ba_5_Ta_4_O_15_, and (c) Cs_3_As_2_Cl_9_. These compounds all exist experimentally.

At this point, we come to an interesting geometric feature of [111]-layered s^2^ halide A_3_B_2_X_9_ phases, their tendency toward a trigonal-pyramidal coordination environment around B, which further rationalizes their stability. To see this, it is helpful to first consider the modes of geometric distortion that often accompany layering. In RP phases, perovskite blocks layered in the [001] direction are separated by AX double layers. For combinations of elements such as Sr–Ti–O and Cs–Pb–I in which A- and X-site charges are equal and opposite, these AX layers are formally neutral, meaning there is no strong driving force pushing ions toward or away from these double layers. In contrast, A_*n*_B_*n*_X_3*n*+2_ compounds layered in the [011] direction have X_2_ double layers, and A_*n*+1_B_*n*_X_3*n*+3_ compounds layered in the [111] direction have AX_3_ double layers. In both cases, these layers separating perovskite blocks are negatively charged, pulling the A- and B-site cations in the perovskite blocks toward these separating layers. This type of distortion can be observed in the experimental structures of oxide and halide perovskites layered in the [011] and [111] directions, as shown in Figure 5a–c.

This shifting of cations toward the interfaces between perovskite regions is an electrostatic driving force for the formation of both [011]- and [111]-layered perovskites. But what is unique to [111]-layered compounds is that when this distortion occurs, the B-site cations adopt three short bonds and three long bonds to neighboring X-site anions, forming motifs resembling trigonal-pyramidal molecules. When viewed through the lens of Lewis structures, in which elements in the same column of the periodic table as nitrogen tend to adopt trigonal-pyramidal coordination, it is not surprising that compounds with arsenic, antimony, or bismuth at the B-site prefer this direction of layering, in which those elements have three nearest neighbors and three more distant neighbors. In fact, this type of distorted octahedral coordination is observed not only in ionic compounds involving arsenic, antimony, or bismuth but also in their elemental solid structures.

**Figure 6:**
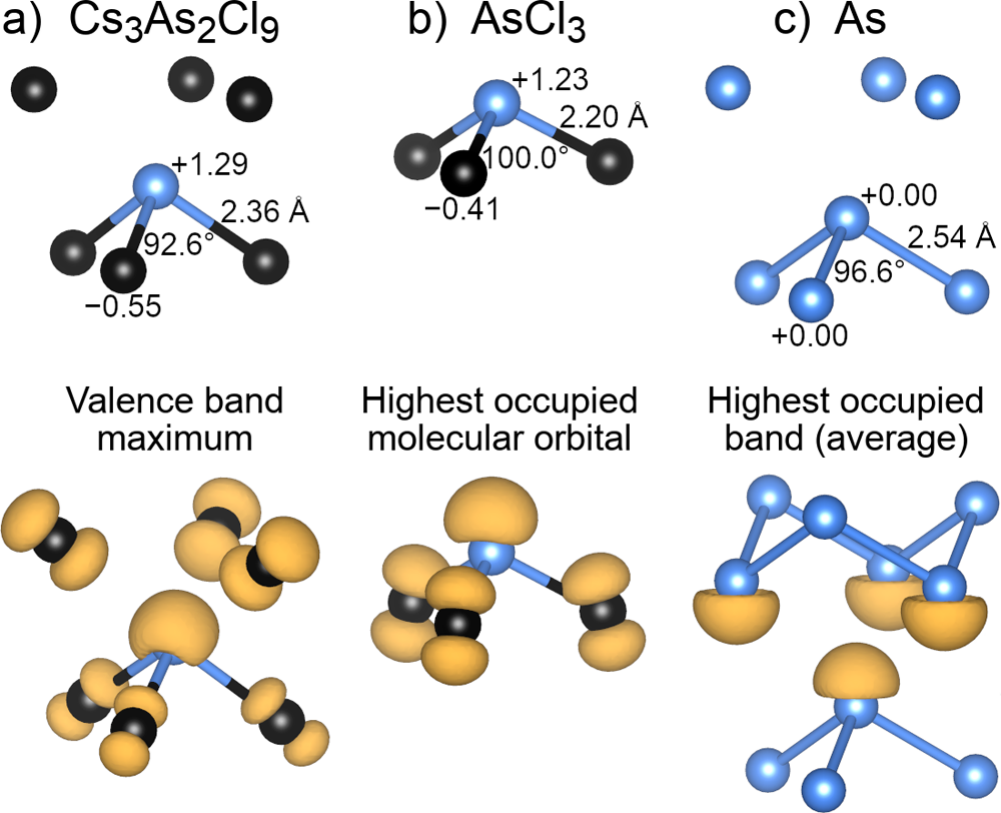
Top panels: coordination environments of arsenic atoms in (a) layered perovskite Cs_3_As_2_Cl_9_, (b) gas-phase AsCl_3_, and (c) elemental solid arsenic. Bonds indicate which atoms are nearest neighbors, while As–Cl bond distances, Cl–As–Cl bond angles, and atomic Bader charges are labeled. Bottom panels: electron densities, shown in orange, correspond to the highest occupied orbitals of the same three structures. Because the three structures are (respectively) an insulator, a molecule, and a metalloid, the orbitals shown are (respectively) a valence band maximum, a highest occupied molecular orbital, and a highest occupied band averaged over all *k*-points.

The distortion of B-site cations away from B–X octahedral centers in [111]-layered s^2^ halide perovskites like Cs_3_As_2_Cl_9_ can therefore be viewed in two different ways: (1) as an electrostatically driven movement of cations toward regions of negative charge and (2) as a covalently driven movement toward trigonal-pyramidal coordination to satisfy the octet rule. In other words, Cs_3_As_2_Cl_9_ can be meaningfully viewed as both Cs_3_^+^As_2_^3+^Cl_9_^–^ and (Cs^+^Cl^–^)_3_(AsCl_3_)_2_. These ionic and covalent driving forces operate synergistically. The importance of the ionic driving force is demonstrated by the fact that these distortions occur in both d^0^ oxides (which lack a B-site lone pair) and s^2^ halides (which have one). The importance of the covalent driving force is demonstrated by the similarities in B–X bond distances and X–B–X bond angles in layered perovskite Cs_3_As_2_Cl_9_, gas-phase AsCl_3_, and elemental solid arsenic, shown in the top panels of Figure 6a–c. The figure also shows the atomic Bader charges within these structures, emphasizing that the arsenic atoms adopt trigonal-pyramidal coordination in cases where they have significant ionic character (Cs_3_As_2_Cl_9_ and AsCl_3_) and in cases where they do not (As).

The bottom panels of Figure 6a–c further show that the highest occupied orbitals of these three arsenic-containing structures also have a similar character. Because of the differing nature of these three structures (an insulator, a molecule, and a metalloid, respectively), their highest occupied orbitals are represented slightly differently in the figure (a valence band maximum, a highest occupied molecular orbital, and a highest occupied band averaged over all *k*-points). In all cases, however, there is significant arsenic lone-pair character in these highest occupied orbitals, as one would expect based on simple Lewis structure arguments. While we typically view the elements in perovskites as being ionic, it is striking to see that the covalent nature of arsenic, antimony, and bismuth—their lone pair that favors trigonal-pyramidal coordination—plays a key role in stabilizing the [111] layering of halide perovskites relative to [011] layering.

## Conclusion

As we have seen, the composition and structure of layered d^0^ oxide and s^2^ halide perovskites have many similarities and some key differences. For both classes of compounds, requirements for charge balance and compatible ionic sizes limit the combinations of elements that could plausibly form layered perovskites. For oxides, many of these plausible compounds indeed form experimentally. For halides, only inorganic perovskites layered in the [111] direction are commonly observed. This is due to not only the features of the layered perovskites themselves but also the stability of other competing phases. Specifically, the absence of [001]- and [011]-layered inorganic halide perovskites is due to the relative stability of the A_4_BX_6_ phase and BX_3_ molecules.

This type of analysis of the driving forces for the formation of layered perovskites helps to highlight the possibilities and challenges in the development of stable new compounds. From one perspective, the restrictions on charge, the destabilizing features of the highest occupied orbitals in halide perovskites, and the preferences of certain elements toward specific B-site coordination environments may be viewed as limiting. However, when this insight is used in support of a synthetic toolkit that allows for the mixing of elements at each crystallographic site, it could help to unlock a greater variety of layered perovskite compounds with potential use in solar energy conversion.

## Data Availability

For all calculations discussed in this paper, the VASP input files (POSCAR, INCAR, and KPOINTS) are available on the authors’ research group GitHub page (https://github.com/bergerlab-wwu).

## Supporting Information Available

DFT pseudopotentials and tabulated numerical results corresponding to figures in the body of the paper (PDF)
