# Supplementary material for: Energetic Stability and Band-Edge Orbitals of Layered Inorganic Perovskite Compounds for Solar Energy Applications
Source: J Phys Chem C Nanomater Interfaces. 2023 Oct 6;127(41):20217–25. doi: 10.1021/acs.jpcc.3c04528 (PMC10591502; doi:10.1021/acs.jpcc.3c04528)
Supplement: Supplementary file 1 — jp3c04528_si_001.pdf [file jp3c04528_si_001.pdf]

Supporting Information for "On the Energetic Stability and Band-Edge Orbitals of Layered Inorganic Perovskite Compounds for Solar Energy Applications"

M. Oluchi Anunobi and Robert F. Berger*

Department of Chemistry, Western Washington University, Bellingham, WA

E-mail: bergerr@wwu.edu

VASP input files. For all calculations discussed in this paper, the VASP input files (POSCAR, INCAR, and KPOINTS) are available on the authors' research group GitHub page (https://github.com/bergerlab-wwu).

Pseudopotentials. In order to produce high-quality results, PBE pseudopotentials in VASP are chosen such that as many electrons as possible are treated as valence. Table S1 shows which electrons are treated as valence.

Table S1: Electrons treated as valence in the VASP PBE pseudopotentials of each of the elements computed in this paper.

Site	Element	Valence electrons
A	Ca	$3s^2 3p^6 4s^2$
	Sr	$4s^2 4p^6 5s^2$
	Ba	$5s^2 5p^6 6s^2$
	Rb	$4s^2 4p^6 5s^1$
	Cs	$5s^2 5p^6 6s^1$
B	Ti	$3s^2 3p^6 4s^2 3d^2$
	Zr	$4s^2 4p^6 5s^2 4d^2$
	Hf	$5s^2 5p^6 6s^2 5d^2$
	Ge	$4s^2 3d^{10} 4p^2$
	Sn	$5s^2 4d^{10} 5p^2$
	Pb	$6s^2 5d^{10} 6p^2$
	As	$4s^2 3d^{10} 4p^3$
	Sb	$5s^2 5p^3$
	Bi	$6s^2 5d^{10} 6p^3$
X	O	$2s^2 2p^4$
	Cl	$3s^2 3p^5$
	Br	$4s^2 4p^5$
	I	$5s^2 5p^5$

Tabulated results. Tables S2 through S8 show the structural and orbital energies of the DFT-PBE-optimized structures discussed throughout in body of the paper.

Table S2: Comparisons of the DFT-PBE structural energy per atom of phases within the Sr-Ti-O, Ca-Ti-O, Cs-Pb-I, and Cs-Ge-I systems, as in Figure 2 in the body of the paper. Phases compared are NaCl-type AX, A_4BX_6 (fully relaxed and with B–X bonds constrained to the corresponding perovskite bond lengths), Ruddlesden-Popper (RP) phases with $n = 1$ and $n = 2$, and cubic perovskite ABX_3.

		AX	A_4BX_6 (relaxed)	A_4BX_6 (constrained)	RP ($n = 1$)	RP ($n = 2$)	ABX_3
Sr-Ti-O	Energy per atom (eV)	−6.0288	−6.8949	−6.8789	−7.4815	−7.7115	−8.0234
	Relative to AX and ABX_3 (eV)	0.0000	0.0405	0.0565	−0.0280	−0.0205	0.0000
Ca-Ti-O	Energy per atom (eV)	−6.4042	−7.1066	−7.0883	−7.5708	−7.7654	−8.0237
	Relative to AX and ABX_3 (eV)	0.0000	0.0338	0.0520	−0.0098	−0.0116	0.0000
Cs-Pb-I	Energy per atom (eV)	−2.7549	−2.8079	−2.8018	−2.7989	−2.8096	−2.8124
	Relative to AX and ABX_3 (eV)	0.0000	−0.0268	−0.0208	−0.0030	−0.0068	0.0000
Cs-Ge-I	Energy per atom (eV)	−2.7549	−2.7921	−2.7843	−2.8079	−2.8162	−2.8196
	Relative to AX and ABX_3 (eV)	0.0000	−0.0078	0.0000	−0.0068	−0.0073	0.0000

Table S3: Results analogous to Table S2, with the key difference that Table S3 includes van der Waals corrections using the D3 method of Grimme. All energies relative to AX and ABX_3 are very similar (within 0.006 eV per atom) with and without van der Waals corrections, justifying our use of DFT without van der Waals corrections throughout the body of the paper.

		AX	A_4BX_6 (relaxed)	RP ($n = 1$)	RP ($n = 2$)	ABX_3
Sr-Ti-O	Energy per atom (eV)	−6.1991	−7.0679	−7.6525	−7.8855	−8.2013
	Relative to AX and ABX_3 (eV)	0.0000	0.0413	−0.0232	−0.0179	0.0000
Ca-Ti-O	Energy per atom (eV)	−6.5777	−7.2787	−7.7332	−7.9299	−8.1902
	Relative to AX and ABX_3 (eV)	0.0000	0.0320	−0.0037	−0.0084	0.0000
Cs-Pb-I	Energy per atom (eV)	−2.9559	−3.0285	−3.0268	−3.0440	−3.0555
	Relative to AX and ABX_3 (eV)	0.0000	−0.0274	0.0003	−0.0051	0.0000
Cs-Ge-I	Energy per atom (eV)	−2.9559	−3.0106	−3.0391	−3.0543	−3.0689
	Relative to AX and ABX_3 (eV)	0.0000	−0.0034	−0.0025	−0.0042	0.0000

Table S4: Structural energies and energy differences per atom of A_4BX_6 phases and combinations of the respective $n = 1$ Ruddlesden-Popper (RP) phases and NaCl-type AX phases, as in Figure 3a in the body of the paper. A_4BX_6 relative energy (i.e., the last column) is defined as the difference between A_4BX_6 and a combination of A_2BX_4 and AX, where a negative value means A_4BX_6 is favored.

	A_4BX_6 energy per atom (eV)	RP ($n = 1$) energy per atom (eV)	AX energy per atom (eV)	A_4BX_6 relative energy per atom (eV)
Ca-Ti-O	−7.1066	−7.5708	−6.4042	0.0400
Sr-Ti-O	−6.8949	−7.4815	−6.0288	0.0584
Ba-Ti-O	−6.8303	−7.4523	−5.9056	0.0596
Ca-Zr-O	−7.1823	−7.6757	−6.4042	0.0311
Sr-Zr-O	−7.0058	−7.6357	−6.0288	0.0455
Ba-Zr-O	−6.9742	−7.6746	−5.9056	0.0571
Ca-Hf-O	−7.6047	−8.3492	−6.4042	0.0372
Sr-Hf-O	−7.4237	−8.3031	−6.0288	0.0523
Ba-Hf-O	−7.3867	−8.3313	−5.9056	0.0625
Cs-Ge-Cl	−3.4088	−3.4648	−3.3196	0.0032
Cs-Ge-Br	−3.1159	−3.1514	−3.0535	−0.0001
Cs-Ge-I	−2.7921	−2.8079	−2.7549	−0.0035
Cs-Sn-Cl	−3.4090	−3.4467	−3.3196	−0.0086
Cs-Sn-Br	−3.1183	−3.1360	−3.0535	−0.0123
Cs-Sn-I	−2.7949	−2.7926	−2.7549	−0.0160
Cs-Pb-Cl	−3.4240	−3.4599	−3.3196	−0.0152
Cs-Pb-Br	−3.1326	−3.1461	−3.0535	−0.0202
Cs-Pb-I	−2.8079	−2.7989	−2.7549	−0.0250

Table S5: Valence band maximum (VBM) energies and energy differences of A_4BX_6 and $n = 1$ Ruddlesden-Popper (RP) phases, as in Figure 3b in the body of the paper. For each compound, the VBM energy is referenced to the energy of the flat, lowest-energy, semi-core band at k-point $\Gamma = (0, 0, 0)$. The difference between A_4BX_6 and A_2BX_4 VBM energies is defined such that a negative value means the VBM of A_4BX_6 is lower in energy.

	A_4BX_6 VBM energy (eV)	RP ($n = 1$) VBM energy (eV)	VBM energy difference (eV)
Ca-Ti-O	56.0263	56.2927	−0.2665
Sr-Ti-O	56.0916	56.2885	−0.1969
Ba-Ti-O	56.3487	56.4843	−0.1356
Ca-Zr-O	46.8364	46.9417	−0.1053
Sr-Zr-O	46.8926	46.9279	−0.0352
Ba-Zr-O	47.1463	47.0845	0.0617
Ca-Hf-O	59.7592	59.8516	−0.0924
Sr-Hf-O	59.8354	59.8598	−0.0244
Ba-Hf-O	60.1271	60.0710	0.0561
Cs-Ge-Cl	23.7049	24.6837	−0.9788
Cs-Ge-Br	23.8660	24.7258	−0.8598
Cs-Ge-I	24.0165	24.6593	−0.6429
Cs-Sn-Cl	20.4271	21.4549	−1.0278
Cs-Sn-Br	20.5854	21.5480	−0.9625
Cs-Sn-I	20.7530	21.6265	−0.8735
Cs-Pb-Cl	19.7234	20.5178	−0.7943
Cs-Pb-Br	20.0048	20.7006	−0.6958
Cs-Pb-I	20.4045	20.9100	−0.5055

Table S6: Results analogous to Table S5, with the key difference that Table S6 includes spin-orbit coupling. All VBM energy differences are very similar (within 0.1 eV per atom) with and without spin-orbit coupling, justifying our use of DFT without spin-orbit coupling in the body of the paper.

	A_4BX_6 VBM energy (eV)	RP ($n = 1$) VBM energy (eV)	VBM energy difference (eV)
Sr-Ti-O	56.1123	56.3630	−0.2507
Cs-Sn-I	21.4383	22.2267	−0.7884
Cs-Pb-I	20.5805	21.1148	−0.5343

Table S7: Comparisons of the DFT-PBE structural energy per atom of phases within the Sr-W-O and Cs-As-Cl systems, as in Figure 4a,b in the body of the paper. Phases compared are NaCl-type AX, [111]-layered $A_3B_2X_9$, [011]-layered ABX_4, and BX_3.

		AX	$A_3B_2X_9$	ABX_4	BX_3
Sr-W-O	Energy per atom (eV)	−6.0288	−7.9863	−8.3004	−9.00876
	Relative to AX and BX_3 (eV)	0.0000	−0.2096	−0.2323	0.0000
Cs-As-Cl	Energy per atom (eV)	−3.3196	−3.3606	−3.3003	−3.2468
	Relative to AX and BX_3 (eV)	0.0000	−0.0826	−0.0292	0.0000

Table S8: Structural energies and energy differences per atom of [011]-layered ABX_4 phases and combinations of the respective [111]-layered $A_3B_2X_9$ phases and AX_3 phases, as in Figure 4c in the body of the paper. ABX_4 relative energy (i.e., the last column) is defined as the difference between ABX_4 and a combination of $A_3B_2X_9$ and AX_3, where a negative value means ABX_4 is favored.

	ABX_4 energy per atom (eV)	$A_3B_2X_9$ per atom (eV)	AX_3 energy per atom (eV)	ABX_4 relative energy per atom (eV)
Ca-W-O	−8.3474	−8.0085	−9.0876	−0.0991
Sr-W-O	−8.3004	−7.9863	−9.0876	−0.0694
Ba-W-O	−8.3080	−8.0519	−9.0876	−0.0260
Rb-As-Cl	−3.2852	−3.3396	−3.2468	0.0337
Rb-As-Br	−2.9229	−2.9837	−2.8341	0.0276
Rb-As-I	−2.5564	−2.6162	−2.4376	0.0201
Rb-Sb-Cl	−3.3235	−3.3670	−3.2338	0.0139
Rb-Sb-Br	−2.9567	−3.0094	−2.8279	0.0124
Rb-Sb-I	−2.5739	−2.6326	−2.4197	0.0114
Cs-As-Cl	−3.3003	−3.3606	−3.2468	0.0350
Cs-As-Br	−2.9416	−3.0100	−2.8341	0.0293
Cs-As-I	−2.5800	−2.6475	−2.4376	0.0208
Cs-Sb-Cl	−3.3492	−3.3942	−3.2338	0.0094
Cs-Sb-Br	−2.9850	−3.0417	−2.8279	0.0092
Cs-Sb-I	−2.6049	−2.6668	−2.4197	0.0070
